# Interplay between Oxidative Stress and Platelet Activation in Coronary Thrombus of STEMI Patients

**DOI:** 10.3390/antiox7070083

**Published:** 2018-07-03

**Authors:** Camilla Calvieri, Gaetano Tanzilli, Simona Bartimoccia, Roberto Cangemi, Alessio Arrivi, Marcello Dominici, Vittoria Cammisotto, Nicola Viceconte, Enrico Mangieri, Giacomo Frati, Francesco Violi

**Affiliations:** 1 Department of Cardiovascular, Respiratory, Nephrology, Anaesthesiology and Geriatric Sciences, Sapienza University of Rome, 00161 Rome, Italy; gaetano.tanzilli@uniroma1.it (G.T.); nicola.viceconte@uniroma1.it (N.V.); enrico.mangieri@uniroma1.it (E.M.); 2 Department of Internal Medicine and Medical Specialties, Sapienza University of Rome, 00161 Rome, Italy; simona.bartimoccia@uniroma1.it (S.B.); roberto.cangemi@uniroma1.it (R.C.); vittoria.cammisotto@uniroma1.it (V.C.); francesco.violi@uniroma1.i (F.V.); 3 Interventional Cardiology Unit, Santa Maria University Hospital, 05100 Terni, Italy; alessio.arrivi@libero.it (A.A.); prof.dominici@gmail.com (M.D.); 4 Department of Medico-Surgical Sciences and Biotechnologies, Sapienza University of Rome, Corso della Repubblica 79, 04100 Latina, Italy; fraticello@inwind.it; 5 Department of AngioCardioNeurology, IRCCS Neuromed, 86077 Pozzilli, Italy

**Keywords:** oxidative stress, STEMI, Thrombolysis in Myocardial Infarction (TIMI) thrombus score, coronary thrombus, platelet activation

## Abstract

Background: Platelet activation and oxidative stress seem to play a key role in coronary thrombus formation and are associated with thrombus burden in ST-elevation myocardial infarction (STEMI). However, the interplay between oxidative stress and platelet activation has not been fully elucidated. Materials and Methods: For 32 patients with STEMI undergoing primary percutaneous coronary intervention (PPCI) and 10 patients with stable angina (SA) and oxidative stress, as assessed by NADPH isoform 2 activity (soluble Nox2-derived peptide, sNox2-dp), levels of oxidized low-density lipoproteins (oxLDLs) and platelet activation markers such as soluble CD40 Ligand (sCD40L) and soluble P-selectin (sP-selectin) were measured in the retrieved material (coronary thrombi plus blood waste) of STEMI patients and in intracoronary blood of SA patients, respectively, and in peripheral blood samples of both groups. Results: In aspirated thrombi and blood waste of STEMI patients we found higher serum levels of sNox2-dp, oxLDLs, sCD40L, and sP-selectin, as compared to the intracoronary blood samples of SA patients. Moreover, in thrombi and blood waste of STEMI patients, a direct correlation between markers of oxidative stress and of platelet activation was found. Also, in STEMI patients a progressive increase of oxidative stress and platelet activation markers was observed according to the thrombus score burden. STEMI patients showed higher peripheral blood Nox2 activity and oxLDL levels as compared to SA patients. Conclusion: This study shows a close relationship between oxidative stress and platelet activation in the intracoronary blood waste and aspirated thrombi of STEMI patients, suggesting a role of oxidative stress in promoting thrombus formation and growth.

## 1. Introduction

Acute coronary thrombosis, arising from atherosclerotic plaque rupture, erosion, or dissection, can result in a total occlusion of a coronary artery and is the main cause of ST-elevation myocardial infarction (STEMI) [1]. The dynamic process of thrombus formation and composition has been extensively studied using thrombus aspiration in primary percutaneous coronary intervention (PPCI) [2]. A growing body of evidence suggests that thrombus formation depends on multiple factors which can act locally and determine its growth and stabilization [3]. Among them, platelet activation and oxidative stress play key roles in atherosclerotic plaque instability and plaque rupture/erosion, with subsequent thrombus formation in STEMI patients [1,3]. In the past, autopsy studies revealed increased levels of cells containing myeloperoxidase (MPO) at the site of instable plaque rupture in patients who died as a result of severe coronary artery disease [4]. Recently, an immunohistochemical study of aspirated thrombi showed the presence of MPO-positive cells in erythrocyte-rich thrombi, which was associated with a greater thrombus size, and demonstrated a close relationship between plasma levels of MPO and oxidized low-density lipoproteins (oxLDLs) [5].

Major players in thrombus formation are oxidized lipids, primarily in the form of oxLDLs, which have been associated to platelet hyperactivity [6]. Furthermore, reactive oxidant species (ROS) via activation of gp91phox (Nox2), the catalytic sub-unit of NADPH oxidase, can also lead to platelet aggregation and thrombus growth [6,7].

Nox2-derived ROS are up-regulated in peripheral blood of STEMI patients [8] but the interplay with platelet activation at coronary site is still unclear.

We aimed to further explore the interplay between oxidative stress (as assessed by sNox2-dp and oxLDLs) and platelet activation (as assessed by sCD40L and sP-selectin). To this end, we analysed the retrieved material (coronary thrombi plus blood waste) of STEMI patients, the intracoronary blood of Stable Angina (SA) patients, and the peripheral blood samples of both groups.

## 2. Materials and Methods

### 2.1. Study Design and Patient Population

Between January 2014 and June 2014, from a prospective observational multicentre study, (Clinical.trial.gov: NCT01090895, approved by Sapienza University of Rome Ethical Committee and registered on 14 January 2010), we retrospectively screened 177 consecutive STEMI patients referred to the catheterization laboratory for PPCI. These patients had undergone manual thrombus aspiration.

Patients were recruited from two centres: (1) the Department of the Heart and Great Vessels “Attilio Reale”, Sapienza-University of Rome; and (2) the Interventional Cardiology Unit, Santa Maria University Hospital, Terni.

Inclusion criteria were: prolonged chest pain (>30 min) with pain onset <12 h, ST segment elevation >0.2 mV in at least two contiguous leads in the initial electrocardiogram (ECG), elevated serum troponin T levels, and successful PPCI (residual coronary stenosis <20%) performed within 12 h of the onset of chest pain and blood sampling prior to PPCI.

Exclusion criteria were: symptom duration >12 h (*n* = 14), rescue PCI (*n* = 16), estimated glomerular filtration rate less than 30 mL/min (*n* = 16), acute infection (*n* = 7), treatment with systemic corticosteroids (*n* = 4), or oral anticoagulants (*n* = 5), or antioxidants (*n* = 5), malignancy (*n* = 3), in-stent thrombosis (*n* = 4), anatomical difficulty in reaching the lesion (*n* = 21), and lack of consent to participate (*n* = 16). Additionally, 34 patients were ineligible because of material absence (*n* = 24) or small size of aspirated samples (*n* = 10). Finally, sufficient thrombotic material (≥1 mm^3^) was retrieved in the 32 patients of study population.

All patients undergoing PPCI received loading doses of aspirin (250–300 mg oral administration), clopidogrel (600 mg oral administration), and unfractionated heparin (1 mg/kg/I.V. (Intra-venous) or 0.7 mg/kg in patients receiving abcximab). None received thrombolysis therapy. PCI of the culprit lesion was performed according to current guidelines [9], and thrombus aspiration was performed prior to angioplasty using a suction catheter (ProntoV3^®^, Vascular Solutions, Minneapolis, MN, USA, or Export^®^ AP, Medtronic, Minneapolis, MN, USA). Intracoronary bolus of glycoprotein IIb/IIIa inhibitors was administered at operator discretion after thrombus retrieval, according to European guidelines [10].

Of 60 consecutive SA patients scheduled for elective diagnostic and/or interventional coronary procedures, 10 patients were enrolled as the control group.

SA was defined as lack of episodes of coronary instability for at least 6 months prior to admission.

In all patients, cardiovascular risk factors were carefully examined, including type II diabetes mellitus (fasting blood glucose >126 mg/dL) or treated diabetes mellitus (intake of a diabetic diet or oral hypoglycaemic agents), dyslipidaemia (low density lipoproteins >130 mg/dL, high density lipoproteins <45 mg/dL, or triglycerides >150 mg/dL, or total cholesterol >200 mg/dL), smoking habit (current and previous habit), family history of early coronary heart disease (CAD) (first degree relative with a history of myocardial infarction <60 years), and hypertension (systolic blood pressure >140 mmHg and/or diastolic pressure >90 mmHg or treated hypertension).

Before the procedure, all patients underwent peripheral blood sampling collection for oxidative stress and platelet activation markers measurements.

The study complied with the Declaration of Helsinki and was approved by the local ethic committees of centres involved.

All patients gave written informed consent before entering the study.

### 2.2. Coronary Angiographic Procedure and Thrombus/Intracoronary Blood Waste Collection

After placement of the guidewire distally to the lesion, the Pronto V3 extraction catheter (Vascular Solutions, Minneapolis, MN, USA) was slowly advanced in aspiration two to five times (depending on operator choice and on the angiographic result obtained) through the culprit lesion. The retrieved materials (coronary thrombi plus blood waste) were collected in a special filter basket. Thereafter, stent delivery balloons were inflated to nominal pressure (pre dilatation or direct stenting were at operator’s discretion). Stent delivery was routinely followed by high-pressure (≥15 atmosphere) balloon inflations with the use of non-compliant or minimally compliant balloons at sizes equivalent to or slightly larger than nominal stent size to achieve residual diameter stenosis ≤20%.

Quantitative coronary angiography and thrombus grade assessment were analysed offline by three independent invasive cardiologists (G.T., N.V., and E.M.) blinded to laboratory data. Thrombus grade was categorized and defined as previously described by Gibson et al. [11].

The intracoronary blood waste was put in ethylenediaminetetraacetic acid (EDTA) tubes and centrifuged. Collected thrombi were immediately washed with saline and fixed with 2% glutaraldehyde in 50 mM Na-cacodylate buffer (pH 7.3). Among SA patients, intracoronary blood samples were picked up directly using the guiding catheter, then collected in EDTA tubes and centrifuged.

### 2.3. Blood Samples and Laboratory Assay

Blood samples were drawn from a peripheral artery (radial or femoral) at baseline (before the start of the procedure). Plasma and serum aliquots were stored at −80 °C in appropriate cuvettes until assay. Complete haemachrome, blood glucose, lipid profile, fibrinogen, creatinine, creatine kinase-MB, and troponin I were evaluated using standard methods. Thrombus aspirates from patients with STEMI were immediately processed. Thrombi were homogenized in 5 mL of a homogenization buffer consisting of 50 mmol L^−1^ Tris-HCl (pH 7.5), 0.25 mol L^−1^ sucrose, 2 mmol L^−1^ tris (2-carboxyethyl) phosphine HCl, 50 mmol L^−1^ NaF, 1 mmol L^−1^ sodium orthovanadate, 10 mmol L^−1^ sodium glycerophosphate, 5 mmol L^−1^ sodium pyrophosphate, Complete Protease Inhibitor Cocktail (Sigma-Aldrich, St. Louis, MO, USA), 1 mmol L^−1^ benzamidine, and 10 mmol L^−1^ phenylmethylsulfonyl fluoride.

Aliquots of homogenate were centrifuged at 13,000× *g* for 10 min. Next, the supernatant was removed and stored at −80 °C until use. Parallel measurements of the same markers were performed in serum from the same patient before the procedure.

### 2.4. Nox2-Derived Peptide (sNox2-Dp)

Nox2-derived peptide, a marker of NADPH oxidase activation, was detected by the ELISA method as previously described [12]. The peptide was recognized by a specific monoclonal antibody against the amino acidic sequence (224–268) of the extra membrane portion of Nox2. Values were expressed as pg/mL. Intra-assay and inter-assay coefficients of variation were 5.2% and 6%, respectively.

### 2.5. Oxidized Low-Density Lipoproteins

Ox-LDLs were measured by commercially available immunoassays (Cusabio, Houston, TX, USA) in the aliquots of plaque homogenate and blood. Intra-assay and inter-assay coefficients of variation were 4.0% and 8.3%. The values were expressed in mU/mL.

### 2.6. Soluble CD40 Ligand and Soluble P-Selectin

Aliquots of plaque homogenate and blood of sCD40L and sP-selectin levels were measured with a commercial immunoassay (DRG International ELISA, Springfield Township, NJ, USA). Intra- and interassay coefficients of variation were 5% and 7% for sCD40L, and 4.3% and 6.1% for sP-selectin, respectively. The values were expressed in ng/mL for sCD40L, and in ng/mL for sP-selectin.

### 2.7. Statistical Analysis

Categorical variables were reported as counts (percentage) and compared with chi-squared test. Continuous variables were first tested for Gaussian distribution with the one-sample Kolmogorov–Smirnov test. They were expressed as mean ± standard deviation (SD), and compared with unpaired Student *t*-test, if normally distributed. They were instead reported as median and first–third quartiles and compared with Mann–Whitney U test if not normally distributed. Correlation analysis was performed with Pearson or Spearman tests, as appropriate. In vitro analysis were also performed using repeated measure ANOVA. Statistical significance was set at the 0.05 level. All tests were 2-tailed, unadjusted for multiplicity, and were performed using SPSS 19 (IBM, Armonk, NY, USA).

## 3. Results

### Clinical, Angiographic and Laboratory Data

We enrolled 32 patients (age 64 ± 12.2 years, 59% males) with STEMI who had undergone manual thrombus aspiration during PPCI, and 10 patients with SA (age 69.8 ± 13 years, 90% males) scheduled for elective PCI. Clinical and demographic characteristics, according to these two groups, are reported in Table 1.

No significant differences in cardiovascular risk factors and therapy on admission were found between the two groups.

**Figure 1:**
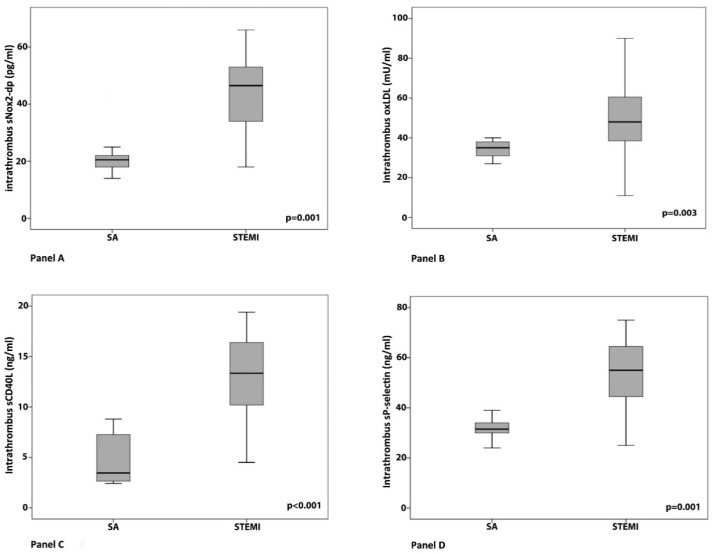
Comparison of intra-coronary levels of sNox2-dp (**A**), oxLDLs (**B**), sCD40L (**C**), and sP-selectin levels (**D**) between patients with stable angina (SA) and with ST-elevation myocardial infarction (STEMI). An unpaired Student *t* test was used for comparison between the two groups. SA: stable angina.

Higher levels of sNox2-dp, oxLDLs, sCD40L, and sP-selectin were found in the intracoronary blood waste and thrombi of STEMI patients as compared to those measured in intracoronary blood samples of SA patients (Figure 1, Panel A–D).

**Figure 2:**
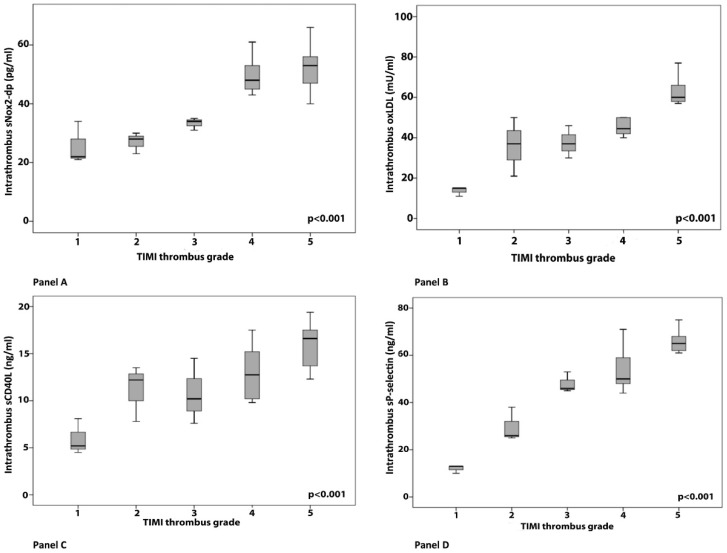
Intra-thrombus levels of sNox2-dp (**A**), oxLDLs (**B**), sCD40L (**C**), and sP-selectin levels (**D**) according to thrombus burden (TIMI thrombus grade) in patients with STEMI. A one-way ANOVA with a post hoc Bonferroni test was used as appropriate to assess the distribution of oxidative stress and platelet activation markers among the study groups. TIMI: Thrombolysis in Myocardial Infarction.

In STEMI patients, a progressive significant increase in sNox2-dp, oxLDL, sCD40L and sP-selectin levels was observed according to progressive higher Thrombolysis in Myocardial Infarction (TIMI) thrombus grade (Figure 2, Panel A–D).

**Figure 3:**
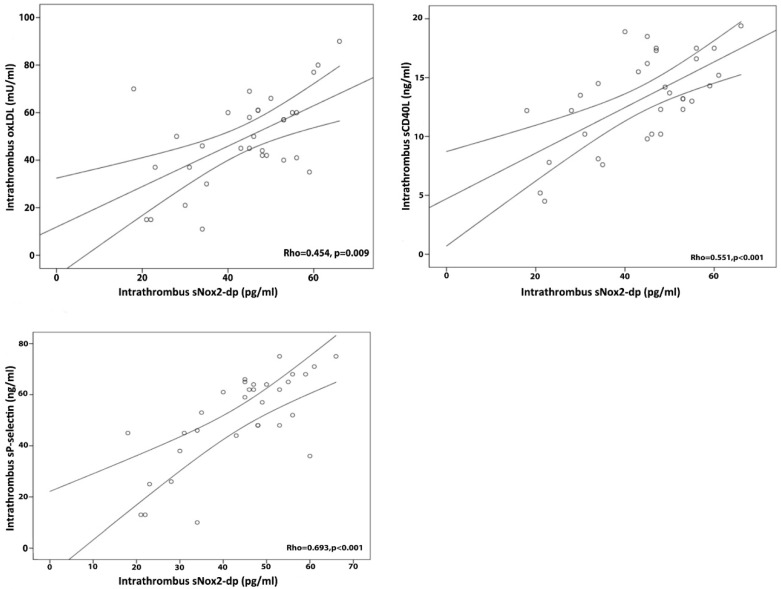
Correlations between intra-thrombus oxidative stress and platelet activation markers. Correlation bivariate analysis was performed with Spearman test. The three lines indicate median values and 95% confidence intervals.

Moreover, in the intracoronary blood waste and thrombi of STEMI patients, sNox2-dp levels correlated directly with oxLDLs (Rho = 0.454, *p* = 0.009), sP-selectin (Rho = 0.693, *p* < 0.001), and sCD40L (Rho = 0.551, *p* < 0.001) levels (Figure 3).

**Figure 4:**
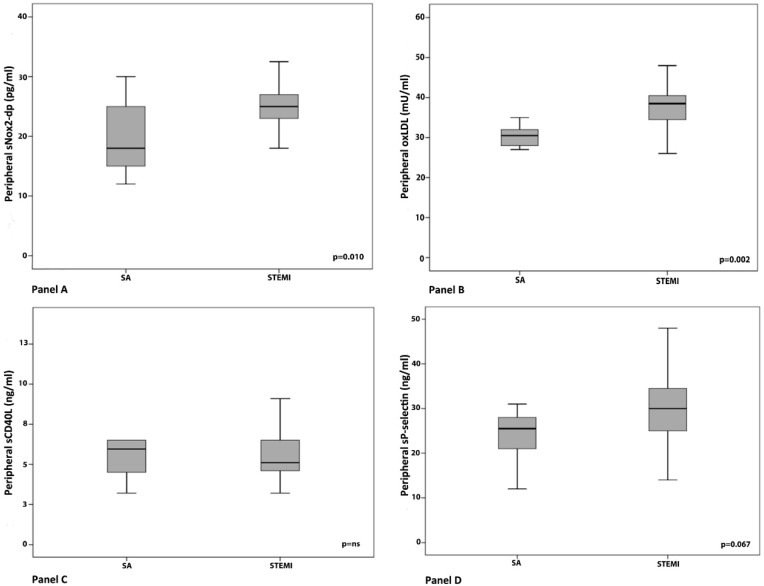
Comparison of pre-PCI peripheral levels of sNox2-dp (**A**), oxLDLs (**B**), sCD40L (**C**), and sP-selectin (**D**) between STEMI and SA patients. An unpaired Student *t* test was used for comparison between the two groups.

In peripheral blood samples obtained before performing PPCI, a significant difference between sNox2-dp and ox-LDL levels was reported when comparing SA with STEMI patients. Regarding platelet activation markers, a trend toward higher sP-selectin levels was found in STEMI patients, while no significant difference in sCD40L levels were found (Figure 4, Panel A–D).

## 4. Discussion

This study showed that oxidative stress and platelet activation are remarkably up-regulated in coronary thrombi of STEMI patients, suggesting a role of ROS in favouring thrombus growth.

Previously, histopathological and immunohistochemical analysis of aspirated thrombi demonstrated that sCD40L and sP-selectin are independent predictors of thrombus fibrin content [5]. Moreover, it has been reported that sP-selectin and sCD40L plasma levels increase during myocardial infarction and after PCI, suggesting that activated platelets might support fibrin accumulation in arterial thrombi [13].

To explore potential mechanisms accounting for platelet activation at site of thrombus growth, we measured markers of oxidative stress and platelet activation in coronary thrombus of STEMI patients. In particular, we focused on Nox2-derived oxidative stress, because patients with hereditary deficiency of Nox2 or mice knockout for the enzyme display ex vivo and in vivo impaired platelet-related thrombus [14,15]. Notably, levels of sNox2-dp and ox-LDLs, markers of oxidative stress, and sCD40L and sP-selectin, markers of platelet activation, were remarkably elevated in coronary thrombi when compared to intracoronary blood of SA patients, suggesting a potential cause–effect relationship between Nox2-derived oxidative stress and thrombosis. However, in intra-thrombus samples of STEMI patients we observed a high variability of the above biomarkers, probably due to the relative content of necrotic core mixed with blood waste. Furthermore, we explored the correlation between markers of oxidative stress and platelet activation, which was detected at the level of coronary thrombi suggesting that oxidative stress may represent a trigger for platelet activation at the site of plaque rupture. This hypothesis was then confirmed by the direct progressive increase of oxidative stress and biomarkers of platelet activation, concomitantly with thrombus burden score.

Recently, a higher content of fibrin, associated to higher levels of sP-selectin, has been reported in coronary thrombi of diabetic STEMI patients [16], but in our study no difference in type 2 diabetes mellitus prevalence was found between the two groups. Previously, the role of sCD40L on ROS generation and on platelet aggregation has been demonstrated [16]. However, its relationship with the subunit Nox2 of NADPH oxidase has never been explored in coronary thrombi. Notably, our results are in agreement with previous report of an up-regulation of Nox2 expression in cardiomyocytes within the infarcted area of patients who died as a result of myocardial infarction [17]. Furthermore, the enhanced levels of ox-LDLs in coronary thrombi observed in the present study could potentially account for Nox2-derived oxidative stress and eventually platelet activation. Previously, we demonstrated that, once generated by activated platelets, oxLDLs propagate platelet activation and increase thrombus growth via Nox2-derived oxidative stress [18]. In addition, Magwenzi et al. reported that oxLDL stimulates the generation of Nox2-derived ROS through a CD36- protein kinase C (PKC) pathway and promotes platelet hyperactivity through modulation of cGMP signalling [19]. Of note, we found no difference in platelet activation markers between STEMI and SA patients in peripheral blood samples before performing PCI. We could speculate that platelet hyper-activation at the coronary site is not reflected in peripheral blood. In this regard, higher levels of platelet activation markers at the coronary site, compared to aorta blood samples, in STEMI and acute coronary syndromes have been found [20,21,22]. However, peripheral blood levels of oxidative stress and platelet activation in SA patients were significantly higher compared with healthy subjects as previously reported by our group [23,24]. Thus, further studies should be performed to explore these triggers of thrombus formation during acute myocardial infarction and to found therapies targeted to lower oxidative stress during acute myocardial infarction.

The study has implications and limitations. Our findings suggest that ox-LDL and Nox2 have a role in promoting thrombus growth, and for this reason larger studies are needed to better elucidate if the down-regulation of these pro-oxidant molecules could hamper thrombus growth.

Another study limitation is the small sample size, and finally, the study does not include a contemporary histopathological characterization of aspirated samples. This hampers the assessment of thrombus age that might provide a further explanation about the time-course interactions between cellular components and biochemical factors.

## 5. Conclusions

Nox2 activity and oxLDLs seem to directly concur with platelet activation at the site of atherosclerotic plaque. The complex interplay between sNox2-dp and oxLDLs could promote platelet activation and thrombus burden growth in coronary thrombi of STEMI patients.

## Figures and Tables

**Table 1:** Clinical characteristics of patients with STEMI and with SA.

	STEMI (*n* = 32)	SA (*n* = 10)	*p*
Age (years) *	64 ± 12.2	69.8 ± 13.0	0.22
Males (%) ^	24 (75)	9 (90)	0.31
Family history of CAD (%) ^	13 (41)	3 (30)	0.55
Obesity (%) ^	14 (44)	2 (20)	0.17
Arterial hypertension (%) ^	24 (75)	6 (60)	0.36
Dyslipidaemia (%) ^	21 (66)	50 (50)	0.37
Type II diabetes (%) ^	10 (31)	3 (30)	0.94
Smoking habits (%) ^	16 (50)	2 (20)	0.09
Previous myocardial infarction (%) ^	6 (19)	4 (40)	0.34
Previous PCI (%) ^	3 (9)	1 (10)	0.95
**Cardiovascular Therapy before PCI**			
Antihypertensive drugs (%) ^	20 (62)	4 (40)	0.79
β-blockers (%) ^	9 (28)	1(10)	0.45
ASA (%) ^	19 (59)	5 (50)	0.87
Statins (%) ^	12 (37)	2 (20)	0.52

**Legend:** Values in () are expressed as percentages, STEMI: ST-elevation myocardial infarction, SA: stable angina; CAD: coronary artery disease; PCI: percutaneous coronary intervention; ASA: Aspirin; ^ chi-squared test; * unpaired Student *t*-test.

## References

[1] Naghavi M., Libby P., Falk E., Casscells S.W., Litovsky S., Rumberger J., Badimon J.J., Stefanadis C., Moreno P., Pasterkamp G. (2003). From vulnerable plaque to vulnerable patient: A call for new definitions and risk assessment strategies: Part I. Circulation.

[2] Yunoki K., Naruko T., Sugioka K., Inaba M., Itoh A., Haze K., Yoshiyama M., Ueda M. (2013). Thrombus aspiration therapy and coronary thrombus components in patients with acute ST-elevation myocardial infarction. J. Atheroscler. Thromb..

[3] Silvain J., Collet J.P., Nagaswami C., Beygui F., Edmondson K.E., Bellemain-Appaix A., Cayla G., Pena A., Brugier D., Barthelemy O. (2011). Composition of coronary thrombus in acute myocardial infarction. J. Am. Coll. Cardiol..

[4] Ferrante G., Nakano M., Prati F., Niccoli G., Mallus M.T., Ramazzotti V., Montone R.A., Kolodgie F.D., Virmani R., Crea F. (2010). High levels of systemic myeloperoxidase are associated with coronary plaque erosion in patients with acute coronary syndromes: A clinicopathological study. Circulation.

[5] Yunoki K., Naruko T., Sugioka K., Inaba M., Iwasa Y., Komatsu R., Itoh A., Haze K., Inoue T., Yoshiyama M. (2012). Erythrocyte-rich thrombus aspirated from patients with ST-elevation myocardial infarction: Association with oxidative stress and its impact on myocardial reperfusion. Eur. Heart J..

[6] Carnevale R., Pignatelli P., Lenti L., Buchetti B., Sanguigni V., Di Santo S., Violi F. (2007). LDL are oxidatively modified by platelets via GP91(phox) and accumulate in human monocytes. FASEB J..

[7] Violi F., Pignatelli P. (2012). Platelet oxidative stress and thrombosis. Thromb. Res..

[8] Niccoli G., Celestini A., Calvieri C., Cosentino N., Falcioni E., Carnevale R., Nocella C., Fracassi F., Roberto M., Antonazzo R.P. (2013). Patients with microvascular obstruction after primary percutaneous coronary intervention show a gp91phox (NOX2) mediated persistent oxidative stress after reperfusion. Eur. Heart J. Acute Cardiovasc. Care.

[9] Kolh P., Windecker S., Alfonso F., Collet J.P., Cremer J., Falk V., Filippatos G., Hamm C., Head S.J., Juni P. (2014). 2014 ESC/EACTS Guidelines on myocardial revascularization: The Task Force on Myocardial Revascularization of the European Society of Cardiology (ESC) and the European Association for Cardio-Thoracic Surgery (EACTS). Developed with the special contribution of the European Association of Percutaneous Cardiovascular Interventions (EAPCI). Eur. J. Cardiothorac. Surg..

[10] Steg P.G., James S.K., Atar D., Badano L.P., Blomstrom-Lundqvist C., Borger M.A., Di Mario C., Dickstein K., Ducrocq G., Task Force on the management of ST-segment elevation acute myocardial infarction of the European Society of Cardiology (ESC) (2012). ESC Guidelines for the management of acute myocardial infarction in patients presenting with ST-segment elevation. Eur. Heart J..

[11] Gibson C.M., de Lemos J.A., Murphy S.A., Marble S.J., McCabe C.H., Cannon C.P., Antman E.M., Braunwald E., Group T.S. (2001). Combination therapy with abciximab reduces angiographically evident thrombus in acute myocardial infarction: A TIMI 14 substudy. Circulation.

[12] Pignatelli P., Carnevale R., Cangemi R., Loffredo L., Sanguigni V., Stefanutti C., Basili S., Violi F. (2010). Atorvastatin inhibits gp91phox circulating levels in patients with hypercholesterolemia. Arterioscler. Thromb. Vasc. Biol..

[13] Sadowski M., Zabczyk M., Undas A. (2014). Coronary thrombus composition: Links with inflammation, platelet and endothelial markers. Atherosclerosis.

[14] Pignatelli P., Carnevale R., Di Santo S., Bartimoccia S., Sanguigni V., Lenti L., Finocchi A., Mendolicchio L., Soresina A.R., Plebani A. (2011). Inherited human gp91phox deficiency is associated with impaired isoprostane formation and platelet dysfunction. Arterioscler. Thromb. Vasc. Biol..

[15] Delaney M.K., Kim K., Estevez B., Xu Z., Stojanovic-Terpo A., Shen B., Ushio-Fukai M., Cho J., Du X. (2016). Differential Roles of the NADPH-Oxidase 1 and 2 in Platelet Activation and Thrombosis. Arterioscler. Thromb. Vasc. Biol..

[16] Sambola A., Ruiz-Meana M., Barba I., Del Blanco B.G., Barrabes J.A., Lip G.Y., Vilardosa U., Sansaloni S., Rello P., Garcia-Dorado D. (2017). Glycative and oxidative stress are associated with altered thrombus composition in diabetic patients with ST-elevation myocardial infarction. Int. J. Cardiol..

[17] Krijnen P.A., Meischl C., Hack C.E., Meijer C.J., Visser C.A., Roos D., Niessen H.W. (2003). Increased Nox2 expression in human cardiomyocytes after acute myocardial infarction. J. Clin. Pathol..

[18] Carnevale R., Bartimoccia S., Nocella C., Di Santo S., Loffredo L., Illuminati G., Lombardi E., Boz V., Del Ben M., De Marco L. (2014). LDL oxidation by platelets propagates platelet activation via an oxidative stress-mediated mechanism. Atherosclerosis.

[19] Magwenzi S., Woodward C., Wraith K.S., Aburima A., Raslan Z., Jones H., McNeil C., Wheatcroft S., Yuldasheva N., Febbriao M. (2015). Oxidized LDL activates blood platelets through CD36/NOX2-mediated inhibition of the cGMP/protein kinase G signaling cascade. Blood.

[20] Majumder B., Koganti S., Lowdell M.W., Rakhit R.D. (2016). Intracoronary platelet and monocyte activation status within platelet-monocyte complexes are determinants of inflammation in ST elevation myocardial infarction1. Clin. Hemorheol. Microcirc..

[21] Gresele P., Falcinelli E., Loffredo F., Cimmino G., Corazzi T., Forte L., Guglielmini G., Momi S., Golino P. (2011). Platelets release matrix metalloproteinase-2 in the coronary circulation of patients with acute coronary syndromes: Possible role in sustained platelet activation. Eur. Heart J..

[22] Ko Y.G., Le V.C., Kim B.H., Shin D.H., Kim J.S., Kim B.K., Choi D., Jang Y., Hong M.K. (2012). Correlations between coronary plaque tissue composition assessed by virtual histology and blood levels of biomarkers for coronary artery disease. Yonsei Med. J..

[23] Loffredo L., Martino F., Carnevale R., Pignatelli P., Catasca E., Perri L., Calabrese C.M., Palumbo M.M., Baratta F., Del Ben M. (2012). Obesity and hypercholesterolemia are associated with NOX2 generated oxidative stress and arterial dysfunction. J. Pediatr..

[24] Carnevale R., Loffredo L., Sanguigni V., Plebani A., Rossi P., Pignata C., Martire B., Finocchi A., Pietrogrande M.C., Azzari C. (2014). Different degrees of NADPH oxidase 2 regulation and in vivo platelet activation: Lesson from chronic granulomatous disease. J. Am. Heart Assoc..

